# Telerehabilitation and onsite rehabilitation effectively improve quality of life, fatigue, balance, and cognition in people with multiple sclerosis: an interventional study

**DOI:** 10.3389/fneur.2024.1394867

**Published:** 2024-08-08

**Authors:** Maria Petracca, Nikolaos Petsas, Giovanni Sellitto, Ilaria Ruotolo, Chiara Livi, Valeria Bonanno, Federica Felicetti, Antonio Ianniello, Serena Ruggieri, Giovanna Borriello, Carlo Pozzilli

**Affiliations:** ^1^Department of Human Neurosciences, Sapienza University of Rome, Rome, Italy; ^2^Department of Public Health and Infectious Diseases, Sapienza University of Rome, Rome, Italy; ^3^Multiple Sclerosis Center, Sant'Andrea Hospital, Rome, Italy; ^4^Neurology Unit, Multiple Sclerosis Center, Fatebenefratelli San Pietro Hospital, Rome, Italy

**Keywords:** telerehabilitation, onsite rehabilitation, multiple sclerosis, quality of life, fatigue, balance, cognition

## Abstract

**Background:**

Telerehabilitation (TR) offers a valuable opportunity to improve access to care and has shown results comparable to onsite rehabilitation (SR) across different conditions. The present study aimed to explore the efficacy of TR and SR in improving clinically meaningful outcomes in people with multiple sclerosis (pwMS).

**Materials and methods:**

Subjects enrolled in the study were assigned to one of two treatment arms: a 6-week TR intervention or a 6-week onsite rehabilitation (SR) intervention. Pre-and post-intervention evaluation included assessment of global wellbeing using the Multiple Sclerosis Quality of Life-54 scale (QoL), fatigue using the Fatigue Severity Status scale (FSS), cognitive status using the Symbol Digit Modalities Test (SDMT), and balance dysfunction using the Berg Balance Scale (BBS). Group-level and single-subject improvements were considered as outcome measures, with QoL as the primary endpoint. To determine significant group changes over time for the entire pwMS cohort, a paired *t*-test was applied to the overall QoL score, focusing on both physical and mental composites. An independent sample *t*-test was used to assess differences in baseline and follow-up performance, as well as changes over time between the intervention groups (TR and SR). This same analysis was repeated for the other clinical domains (FSS, BBS, and SDMT). The minimal clinically important difference (MCID) according to treatment group (TR vs. SR) was explored using logistic regression. Additionally, a multiple linear regression model was applied to evaluate the impact of baseline clinical-demographic features on the observed post-intervention modifications.

**Results:**

A total of 51 subjects completed the study (37 women, mean age 46.3 ± 9.8, median Expanded Disability Status Scale 3.5, min. 2, max. 6.5). The entire sample benefited from the rehabilitation treatment, with significant improvements observed at both the group and individual levels across all measured domains for both intervention groups (TR vs. SR). Quality of life improved significantly (p = 0.005), as did fatigue and balance (both p < 0.001), and cognition (p = 0.003).

**Conclusions:**

Both SR and TR approaches effectively improved the perception of fatigue, cognitive performance, balance, and quality of life in a population of MS patients with moderate disability.

## 1 Introduction

Neurorehabilitation plays a well-established role in Multiple Sclerosis (MS) management (1). However, it poses complex challenges in healthcare delivery, requiring access to specialized centers and the allocation of dedicated time, resulting in high direct and indirect costs (2). In this context and, more recently, during the COVID-19 pandemic, telemedicine has been proposed as a viable and reliable solution for outpatients' needs (3). Telerehabilitation (TR) offers great promise in improving access to rehabilitation care (4, 5), and the increasing diffusion of technology promotes its application.

From an efficacy viewpoint, TR appears to yield similar results to onsite rehabilitation (SR) across conditions (6–8). In MS, moderate-quality evidence suggests that physical therapeutic modalities improve quality of life (QoL), fatigue, mobility, and cognition (9, 10). However, there is still insufficient evidence to support the effectiveness of remotely supervised physical therapy in these domains (7, 11, 12). To date, studies have mainly focused on motor outcomes such as balance, arm/hand function, walking speed, and muscle strength (13–17). Only one study has explored the impact of remotely supervised physical therapy on QoL and fatigue (18), and, to the best of our knowledge, no data are available on its effectiveness on cognitive performance.

Additionally, no analyses exploring improvements at the single-subject level have been conducted.

Therefore, this study explored the efficacy of remotely supervised physical therapy (referred to as TR) compared to SR on patient-reported outcomes (PRO) and performance measures of quality of life (QoL), fatigue, balance, and cognition. We attempted to clarify the impact of TR on global wellbeing and symptoms that, together with motor disability, contribute the most to patients' perception of health (19–21).

The effectiveness of the rehabilitative intervention was evaluated not only at the group level but also by exploring improvements at the single-subject level. Finally, we investigated the impact of baseline clinical-demographic features on clinical outcomes of pwMS undergoing TR and SR to identify features that might guide the adoption of a specific approach in individual cases.

## 2 Materials and methods

### 2.1 Study design

This single-center interventional study evaluated the impact of a 6-week TR intervention vs. a 6-week SR intervention on clinical and behavioral parameters in subjects with MS. The study was conducted in accordance with the ethical code of the ethics committee of Sapienza University of Rome (number 5416, approval date 21/11/2019). All patients provided written informed consent before participating in the study. The study adhered to good clinical practice and the principles of the Declaration of Helsinki.

### 2.2 Patients

Subjects with a diagnosis of MS according to the revised McDonald Criteria 2017 (22) and an Expanded Disability Status Scale (EDSS) score (23) between 2.0 and 6.5 were prospectively enrolled at the MS center of Sant'Andrea Hospital in Rome. Due to potential safety and adherence concerns, the study did not include patients with significant cardiopulmonary problems, cognitive disorders, and severely impaired walking ability (EDSS >6.5). Clinical stability at study entry was ensured to avoid potential interpretation bias in the study findings, excluding patients with relapses, pharmacological treatment modifications, or rehabilitation treatment within the 4 weeks before the screening visit.

### 2.3 Study procedures

Enrolled subjects were assigned to one of two treatment arms: a 6-week TR intervention or a 6-week SR intervention. Due to the COVID-19 pandemic-related restrictions, all subjects enrolled between October 2020 and November 2021 (*N* = 31) were assigned to the TR intervention, which was compatible with the ongoing restrictions and the need to provide treatment. Once it became possible to grant hospital access for rehabilitation (December 2021), enrolled subjects were assigned to the SR group to reach the established minimum sample size (*N* = 25). After screening and enrollment, subjects were followed up for 8 weeks.

Clinical evaluation was performed at the screening/baseline visit, 1 week prior to the initiation of the 6-week program, and within 1 week after its conclusion by neurologists and physical therapists who were blinded to the patients' allocation. All aspects identified as key for the definition and perception of health in pwMS (global wellbeing, pain, cognitive and balance dysfunction, and fatigue) (19–21, 24) were assessed using patient-reported outcomes (PROs). Domains that could not be explored with PROs due to the lack of validated instruments (balance and cognitive dysfunction) were objectively evaluated with clinician-administered performance measures, specifically the Berg Balance Scale and the Symbol Digit Modalities Test. Each visit included the following:

- **Multiple Sclerosis Quality of Life-54 scale (MSQL-54)**: This scale is used for the multidimensional self-evaluation of global wellbeing, including physical function, physical and emotional limitations, pain, emotional wellbeing, energy, health perceptions, social function, cognitive function, health distress, overall quality of life, sexual function, and sexual satisfaction (25). The 54 items are organized into 12 multi-item and two single-item subscales. All items inquire about QoL over the preceding month, except item 2 (Change in Health), which refers to the preceding year. The resulting scores range from zero to 100. Two weighted composite scores (Physical Health Composite and Mental Health Composite) are derived by combining scores of the relevant subscales. Higher scores correspond to better QoL.
- **The Fatigue Severity Status Scale (FSS)**: This scale is used to evaluate the subjective perception of fatigue (26). The FSS is a 9-item scale that measures the severity of fatigue and its effect on a person's activities and lifestyle. Answers are scored on a 7-point scale where 1 = strongly disagree and 7 = strongly agree, with a minimum possible score of 9 and the highest possible score of 63. Higher scores indicate more severe fatigue and a greater impact on the person's activities.
- **The Berg Balance Scale (BBS)**: This scale is used to evaluate functional balance and the risk of falling (27). The BBS is a 14-item list, with each item consisting of a five-point ordinal scale ranging from 0 to 4, with 0 indicating the lowest and 4 indicating the highest level of function. Lower scores correspond to a higher risk of falling.
- **The Symbol Digit Modalities Test (SDMT)**: This test is used to assess higher-level cognitive functions (28). Using a reference key, the examinee has 90 s to pair specific numbers with given geometric figures. Scoring involves summing the correct substitutions within the 90-s interval, with a maximum score of 110. Higher scores correspond to better performance.

Baseline evaluation also included collecting demographic data [age, sex, education, body mass index (BMI)], clinical history (disease duration, current and past therapy), and EDSS rating to evaluate global disability.

### 2.4 Rehabilitation interventions

The basic structure of the TR program consisted of 18 one-on-one remotely supervised physical treatment sessions conducted via interactive, whole-body view videoconference over 6 consecutive weeks (average: 3 sessions/week, 45-min long). To maximize the effectiveness of the videoconference modality, we used the XBOX One gaming console (Microsoft Inc., Redmont, USA), a commercial game console designed for simplicity and ease of use. It was equipped with a Kinect sensor peripheral (v2), which provides an optimal field and angle of vision, resolution, and audio. The kit, consisting of the Kinect for XBOX One sensor and the XBOX One gaming console (https://www.microsoft.com/en-us/store/b/xboxconsoles?icid=XboxCat_QL1_Consoles), was delivered to the patient pre-configured with Skype for Kinect and a corresponding online account. Upon delivery, which occurred during the baseline clinical visit, a 15-min training session was conducted to ensure each participant could assemble and use the console proficiently.

The SR program consisted of 18 one-on-one, in-person, supervised physical treatment sessions and was similar to the TR program in terms of duration (6 weeks), frequency (three sessions per week, each 45-min long), and mode (individual interaction between physiotherapist and patient). Subjects allocated to the SR program attended as outpatients.

In both cases (TR and SR), the rehabilitation intervention was targeted to the patient's specific neurological deficits. It included a combination of both aerobic and anaerobic exercises, balance exercises, core stability, flexibility, and mobility aimed at increasing muscle strength, recruiting less competent districts, decreasing spasticity, improving balance, restoring correct gait patterns, and improving functional activities of daily living. The programs incorporated a progressive overload scheme with high-intensity interval training and a motor learning approach in the more advanced stages. Specifically, each session included 15 to 20 min of endurance training according to the patient's tolerance, followed by muscle power training (four to eight exercises with intensity ranging from 1 to 4 sets of 8 to 15 repetitions each). Within each session, training began with the largest muscle groups, prioritizing the lower limbs, with rest intervals ranging from 2 to 4 min based on individual needs. Endurance and power training were followed by task-oriented training, structured into semi-dynamic and dynamic phases. In the semi-dynamic phase, exercises with external and internal feedback were conducted, while the dynamic phase focused on walking and target-achievement exercises. During task-oriented training, rest intervals varied according to the individual fatigue levels. Equipment included a treatment table/yoga mat, a Bobath football ball (65 cm in diameter), weights (1, 2, and 3 kg), and elastic bands with increasing resistance. Equipment usage varied throughout the 6-week training period according to individual progression.

The initial stages involved static balance exercises on the table mat. Once the patient improved in body perception and motor control, they were moved to the yoga mat, performing exercises in different positions with progressively narrowed support bases, eventually adding unstable surfaces in the more advanced stages. The final stages included dynamic balance exercises performed in dual-task and task-oriented modalities. Muscle activation exercises were always preceded and followed by stretching sessions, with priority in progression given to muscle and core districts, first with gravitational involvement on the mat and then with antigravitational participation on both the tab and the yoga mat. In the advanced stages of the program, aerobic exercises such as walking and step simulation were introduced, reducing rest interval times and introducing explosive exercises such as squats and jumps whenever possible.

All the treatment steps just described were performed using proprioceptive neuromuscular facilitation (PNF). PNF is an applied technique designed to promote the response of the neuromuscular system, improving mobility, muscular strength and endurance, joint stability, balance, and neuromuscular control by stimulating the proprioceptors within the skin, joints, muscles, and tendons (29). PNF techniques selected for this rehabilitation protocol included the relaxation-contraction method and the relaxation-contraction-antagonist method to improve mobility, the repetitive contraction method and the replication method to facilitate neuromuscular outputs, as well as the relaxation-contraction and relaxation-contraction-antagonist methods to enhance performance in power dominant and endurance dominant tasks (30).

### 2.5 Outcome measures

At the group level, we considered longitudinal changes in clinical scales computed as the difference (delta) between follow-up and baseline test scores. Additionally, following recommendations from contemporary statistical guidelines (31, 32), we aimed to provide an interpretation of study findings based on clinical relevance at the individual level. Single-subject improvements in QoL, fatigue, balance, and cognition were estimated by applying the published cut-off for the definition of minimal clinically important difference (MCID): 5 points for MSQoL54, 0.45 points for FSS, 3 points for BBS, and 6 points for SDMT (33–36).

### 2.6 Endpoints

The primary endpoint was to compare QoL changes in pwMS undergoing TR vs. SR (group mean differences).

The secondary study endpoints were as follows:

- to compare changes in fatigue, balance, and cognition in pwMS undergoing TR vs. SR (group mean differences);
- to evaluate differences in the frequency of subjects showing MCID across different clinical domains in pwMS undergoing TR vs. SR; and
- to evaluate the impact of baseline clinical-demographic features and fatigue on longitudinal changes across different domains in pwMS undergoing TR vs. SR (both at the group and individual levels).

### 2.7 Sample size estimation

Although a formal estimation of sample size was not conducted, a minimum sample size of 25 participants per arm was established, which is in line with previous studies exploring the impact of exercise on QoL in MS patients (37).

### 2.8 Statistical analysis

Baseline clinical and behavioral characteristics were evaluated and compared between intervention groups (TR vs. SR) with the Student's *t*-test with equal variance assumption, the Mann–Whitney test, or chi-square with continuity correction, as appropriate.

Each measure underwent descriptive statistics and evaluation of the normality of the distribution (through the Kolmogorov–Smirnov testing), both for the whole cohort and within the group and time point.

For the primary endpoint, a paired *t*-test was applied to the overall QoL score and physical and mental composites to determine significant group changes over time for the entire pwMS group. The effect size was evaluated using Hedge's g, calculated as the mean difference between sample 1 and sample 2 divided by the pooled variance, with bias correction. Effect sizes were interpreted as small (0.2), medium (0.5), and large (0.8). An independent sample *t*-test was applied to assess differences in baseline and follow-up performance and changes over time between the intervention groups (TR and SR).

The same analysis was repeated for the other clinical domains (FSS, BBS, and SDMT). The significance level was set at a *p* < 0.008, Bonferroni corrected for multiple comparisons (0.05/6, as the number of tested outcomes).

The difference in MCID frequency across different clinical scales in the two groups (TR vs. SR) was preliminary tested with Fisher's exact test and further explored via logistic regression with MCID as dependent variables, sex, age, BMI, baseline EDSS, and FSS as adjustment covariates, with the treatment group (TR vs. SR) as covariates of interest. BMI, baseline EDSS, and FSS were included as adjustment covariates to account for their possible impact on performance during rehabilitation. The significance level was set at a *p* < 0.008, Bonferroni corrected for multiple comparisons (0.05/6, as the number of tested outcomes).

To evaluate the impact of baseline clinical-demographic features on the observed post-intervention modifications, a multiple linear regression model was applied. The measured change (computed as delta: follow-up score—baseline score) was the predicted value, and the baseline measure value was considered an adjustment covariate, along with age, sex, BMI, baseline EDSS, and FSS. The intervention group was entered as a covariate of interest. BMI, baseline EDSS, and FSS were included as adjustment covariates to account for their possible impact on performance during rehabilitation. The model was implemented following the European Medicines Agency guidelines on longitudinal interventional trials. The significance level was set at a *p* < 0.008, Bonferroni corrected for multiple comparisons (0.05/6, as the number of tested models).

## 3 Results

### 3.1 Study population

A total of 61 pwMS were recruited between October 2020 and November 2022. Of these, 10 participants (16.4%) discontinued the study: eight participants from the TR group (five participants due to Internet connectivity issues, two participants due to intervening medical conditions, one participant due to non-compliance), and two participants from the SR group (one participant due to an intervening medical condition and one participant due to non-compliance). A total of pwMS (83.6%) completed the study.

### 3.2 Demographics and baseline characteristics

Baseline demographic and clinical characteristics of the study population, both as a whole group and stratified by intervention type (TR vs. SR), are reported in Table 1. There were no significant differences in the reported baseline characteristics between the SR and TR groups. Additionally, no relapses occurred over the study period.

**Table 1:** Baseline demographic and clinical characteristics in the entire population and according to treatment group.

	**All pwMS (*n* = 51)**	**SR (*n* = 26)**	**TR (*n* = 25)**	***p*-value**
Age, years	46.31 ± 97.79	45.92 ± 10.94	46.72 ± 8.64	0.775
Sex (F/M)	37/14	18/8	19/6	0.820
Disease duration, years	14.16 ± 10.06	12.21 ± 9.63	16.19 ± 10.30	0.161
Education, years	13.76 ± 3.32	13.00 ± 3.50	14.56 ± 2.99	0.094
BMI	21.91 ± 2.68	21.80 ± 2.62	22.03 ± 2.80	0.764
EDSS (median [range])	3.5 [2.0–6.5]	3.75 [2.0–6.5]	3.5 [2.5–6.5]	0.954
Treatment (none/L-MET/HET)	10/32/9	5/16/5	5/16/4	0.092

All values are reported as mean ± standard deviation unless otherwise indicated. The reported p is derived from the between-group comparison using either a student t-test with equal variance assumption, the Mann-Whitney test, or a chi-square with continuity correction, as appropriate. BMI, body mass index; EDSS, expanded disability status scale; F, females; HET, high efficacy therapy (including natalizumab and anti-CD20); L-MET, low-moderate efficacy therapy (including interferons, glatiramer acetate, teriflunomide, dimethyl fumarate, S1P inhibitors, cladribine); M, males; pwMS, people with multiple sclerosis; SR, onsite rehabilitation; TR, telerehabilitation.

### 3.3 Impact of the rehabilitation interventions on QoL

Overall QoL, as well as physical and mental composites, showed a low to moderate improvement over time (Bonferroni corrected for multiple comparisons at a *p* < 0.008), with no difference between TR and SR (Tables 2, 3).

**Table 2:** Quality of life before and after rehabilitation in the entire population and according to treatment group.

	**Time**	**All pwMS**	**SR**	**TR**	***p*-value (T0 vs. T1)**	Hedge's g^*^(T0 vs. T1)	***p*-value (SR vs. TR)**
Overall QoL	0	58.33 ± 16.56	54.87 ± 16.67	61.93 ± 15.97			0.129
	1	62.29 ± 15.78	60.32 ± 17.51	64.62 ± 13.49	0.005	0.29	0.353
MHC	0	56.20 ± 20.32	52.86 ± 21.15	61.37 ± 20.58			0.157
	1	64.68 ± 18.98	61.15 ± 19.97	68.83 ± 17.27	0.002	0.43	0.165
PHC	0	50.16 ± 14.97	49.72 ± 14.30	52.32 ± 17.25			0.566
	1	58.45 ± 16.59	59.22 ± 15.84	57.73 ± 17.48	< 0.001	0.52	0.761

All values are reported as mean ± standard deviation. The reported p for T0 vs. T1 is derived from the within-group comparison using a paired-sample t-test. The reported p for SR vs. TR is derived from the between-group comparison using an independent-sample t-test. g^*^, corrected g; MHC, Mental Health Composite; PHC, Physical Health Composite; pwMS, people with multiple sclerosis; QoL, quality of life; SR, onsite rehabilitation; T0, baseline; T1, post-intervention follow-up; TR, telerehabilitation.

**Table 3:** Longitudinal variations in quality of life before and after rehabilitation in the entire population and according to treatment group.

	**All pwMS**	**SR**	**TR**	**p-value (SR vs. TR)**
Overall QoL change	4.69 ± 10.92	5.45 ± 11.86	3.79 ± 9.90	0.604
MHC change	8.47 ± 17.53	8.19 ± 16.59	8.79 ± 18.98	0.908
PHC change	8.29 ± 13.66	9.33 ± 12.77	7.06 ± 14.87	0.580

All values are reported as mean ± standard deviation. The reported p for SR vs. TR is derived from the between-group comparison using an independent-sample t-test. MHC, Mental Health Composite; PHC, Physical Health Composite; pwMS, people with multiple sclerosis; QoL, quality of life; SR, onsite rehabilitation; TR, telerehabilitation.

In the entire study population, 22 patients showed an MCID in overall QoL, 24 patients in the QoL physical composite, and 27 patients in the QoL mental composite, with no differences between SR and TR (overall QoL: 13 vs. 9, *p* = 0.770; QoL physical composite: 14 vs. 10, *p* = 0.768; QoL mental composite: 17 vs. 10, *p* = 0.244). The odds ratio for the TR vs. SR group was 0.766 (95% C.I. 0.185–3.175) for overall QoL (*p* = 0.713), 0.599 (95% C.I. 0.145–2.470) for the QoL physical composite (p = 0. 478), and 0.558 for the QoL mental composite (95% C.I. 0.110–2.820) (*p* = 0.480).

### 3.4 Impact of the rehabilitation interventions on fatigue, balance, and cognition

All outcome measures showed a low to moderate improvement over time (Bonferroni corrected for multiple comparisons at a *p* < 0.008), with no difference between TR and SR (Tables 4, 5). In the entire study population, 13 patients showed an MCID in fatigue (1/11/1 respectively showing mild/moderate/severe fatigue at baseline), 26 patients in balance (11 showing risk of falling at baseline), and 16 patients in cognition (eight patients showing baseline performance below normative values), with no differences between SR and TR (fatigue 7 vs. 6, *p* = 1; balance 11 vs. 15, *p* = 0.171; cognition 7 vs. 9, *p* = 0.542).

**Table 4:** Fatigue, balance, and cognition before and after rehabilitation are present in the entire population and according to the treatment group.

	**Time**	**All pwMS**	**SR**	**TR**	**p-value (T0 vs. T1)**	Hedge's g^*^(T0 vs. T1)	**p-value (SR vs. TR)**
FSS	0	47.42 ± 9.96	47.54 ± 10.14	47.29 ± 9.98			0.931
	1	42.02 ± 10.88	41.08 ± 9.63	43.16 ± 11.98	< 0.001	0.51	0.496
BBS	0	49.08 ± 6.17	49.50 ± 6.42	48.62 ± 5.99			0.621
	1	53.20 ± 4.57	52.96 ± 5.42	53.46 ± 3.53	< 0.001	0.75	0.705
SDMT	0	35.52 ± 10.78	36.50 ± 10.51	34.46 ± 11.20			0.510
	1	39.48 ± 11.10	38.81 ± 11.01	40.21 ± 11.39	0.003	0.33	0.660

All values are reported as mean ± standard deviation. The reported p for T0 vs. T1 is derived from the within-group comparison using a paired-sample t-test. The reported p for SR vs. TR is derived from the between-group comparison using an independent-sample t-test. BBS, Berg Balance Scale; FSS, Fatigue Severity Status scale; g^*^, corrected g; pwMS, people with multiple sclerosis; SDMT, symbol digit modalities test; SR, onsite rehabilitation; T0, baseline; T1, post-intervention follow-up; TR, telerehabilitation.

**Table 5:** Longitudinal variations in fatigue, balance, and cognition before and after rehabilitation in the entire population and according to treatment group.

	**All pwMS**	**SR**	**TR**	***p*-value (SR vs. TR)**
FSS change	−5.40 ± 8.65	−6.46 ± 8.85	−4.25 ± 8.47	0.372
BBS change	3 ± 9.35	3.46 ± 5.01	4.83 ± 4.75	0.327
SDMT change	3.61 ± 7.93	2.31 ± 4.43	5.09 ± 10.52	0.225

All values are reported as mean ± standard deviation. The reported p for SR vs. TR is derived from the between-group comparison using an independent-sample t-test. BBS, Berg Balance Scale; FSS, Fatigue Severity Status scale; pwMS, people with multiple sclerosis; SDMT, symbol digit modalities test; SR, onsite rehabilitation; TR, telerehabilitation.

The odds ratio for the TR vs. SR group for fatigue was 0.942 (95% C.I. 0.241–3.682) (*p* = 0.932), 4.161 (95% C.I. 0.656–26.408) for balance (*p* = 0. 130), and 1.218 (95% C.I. 0.320–4.643) for cognition (*p* = 0.772).

### 3.5 Influence of baseline features on clinical outcomes

Among clinical-demographic features, baseline scores for physical health composite, mental health composite, BBS, and SDMT significantly predicted changes in their respective outcome measures over time (Table 6). *P* values remained significant after Bonferroni correction for multiple comparisons at a *p* < 0.008. BMI showed a trend toward association with QoL outcomes, but this did not survive Bonferroni correction. The regression analysis showed no significant impact of the intervention group or any other baseline variable on clinical outcomes (Table 6).

**Table 6:** Multiple regression models evaluate the impact of baseline features, fatigue change, and treatment group on clinical outcomes change over time.

	**Model**				**Predictors**			
	**Adj R** ^2^	**F**	**DF**	***p*** **-value**		**Standardized beta**	***t*** **-value**	***p*** **-value**
Overall QoL change	0.183	2.467	7-39	0.034	BMI	0.371	2.280	0.028
MHC change	0.309	3.933	7-39	0.002	Baseline MHC	−0.616	−4.180	< 0.001
					BMI	0.318	2.085	0.044
PHC change	0.211	2.678	7-37	0.024	Baseline PHC	−0.581	−2.830	0.007
					BMI	0.476	2.678	0.011
FSS change	0.059	2.511	6-43	0.198	Baseline FSS	−0.322	−2.282	0.028
BBS change	0.460	6.850	7-41	< 0.001	Baseline BBS	−0.772	−5.696	< 0.001
SDMT change	0.149	2.180	7-40	0.057	Baseline SDMT	−0.512	−3.227	0.002

BBS, Berg Balance Scale; FSS, Fatigue Severity Status scale; MHC, Mental Health Composite; PHC, Physical Health Composite; pwMS, people with multiple sclerosis; QoL, quality of life; SDMT, symbol digit modalities test; SR, onsite rehabilitation; T0, baseline; T1, post-intervention follow-up; TR, telerehabilitation.

The logistic models predicting MCID produced similar results:

- Mental health composite MCID: baseline mental health composite score (OR 0.904, 95% C.I. 0.847–0.966, *p* = 0.003);
- BBS MCID: baseline BBS score (OR 0.584, 95% C.I. 0.409–0.836, *p* = 0.003);
- SDMT MCID: baseline SDMT score (OR 0.915, 95% C.I. 0.841–0.995, *p* = 0.037).

## 4 Discussion

Our results support the efficacy of TR on QoL, fatigue, balance, and cognition in pwMS. Indeed, TR showed similar effects to SR, both at the group and individual levels. The effect sizes for QoL improvement post-rehabilitation are in agreement with previously reported data, showing low to moderate effects (37). The highest effect size was observed in the physical composite score, which is in line with the nature of the intervention. QoL is an ideal outcome in MS, serving as a meaningful indicator of disease impact on daily functioning, treatment success, and future progression (24). Despite the clinical significance of QoL and the potential for significant improvements in QoL through aerobic exercise and physiotherapy (37), such interventions are often hindered in clinical practice by logistical difficulties.

The initial attempts to use remote connections for QoL improvement involved web-based training and behavioral interventions, which either lacked efficacy or showed improvements limited to the physical component of QoL (13, 15, 17, 38, 39). In contrast, our one-on-one, real-time videoconferencing approach also significantly improved overall QoL and the QoL mental composite, with no detectable difference compared to a standard SR approach.

Indeed, real-time interaction via videoconferencing is an effective modality among TR approaches, enabling the development of a strong therapeutic relationship with the therapist and providing real-time feedback and support from a healthcare professional, likely producing effects beyond mobility benefits alone. Supporting this interpretation, a recent study using virtual reality TR with asynchronous communication and feedback between the therapist and the patient only improved the QoL physical composite score (40).

The relevance of therapist–patient interaction for achieving non-physical outcomes is further highlighted by a previous study applying TR in pwMS with high disability levels (5.5 < EDSS>7.5), which showed significant group improvements in pain and cognitive QoL items compared to a control group receiving periodic newsletters (37). In this study, the lack of physical benefit was probably related to the high level of disability of the enrolled population (41).

Our findings regarding fatigue, balance, and cognition align with the results described for QoL at both the group and individual levels. Regarding fatigue, previous studies have shown some beneficial but inconsistent effects of e-training and behavioral interventions (13, 17, 39), possibly depending on the baseline level of fatigue of the enrolled populations. Indeed, the presence of a clinical deficit is a necessary prerequisite for its improvement after rehabilitation (17).

Conflicting results regarding improvement in static and dynamic balance have been obtained to date, as assessed via BBS following asynchronously supported web-based exercise programs (13, 15, 16). Specifically, significant improvements were only identified for programs that included balance-tailored exercises (13). Finally, the post-intervention improvement of cognitive function observed in this study, although small, is similar to the one reported after home-based cognitive training [see Di Tella et al. (42) for a comprehensive review] or behavioral intervention promoting physical activity (39).

As for the mechanisms behind the observed clinical improvements, both motor and cognitive rehabilitation are believed to modulate neuroplasticity, inducing cortical reorganization, functional rearrangements of neural connections, and changes in the microstructural properties of the white matter (43). Beyond group-level results, in our population, the physiotherapy intervention was also able to induce individually meaningful improvements in 25% to 53% of pwMS across different domains, with no differences between the two approaches, suggesting that both might be valuable instruments to address clinically meaningful outcomes for pwMS.

Our findings also suggest that improvements in specific domains, both at the group and individual levels, are affected by the relative level of impairment at baseline in pwMS. This was true for all explored outcomes except for fatigue, which is consistent with previous data showing the independence of fatigue change over time from baseline features (44).

The fact that improvements are more difficult to achieve in people with a higher level of performance, at least in a population with a moderate level of motor disability, should be taken into account when designing patient-tailored interventions to set realistic goals. Despite our encouraging results, we would like to acknowledge possible barriers to implementing TR, such as low technological literacy (5).

In our population, the dropout rate would have been essentially the same in the TR and SR groups if not for the patients discontinuing the program due to connectivity issues. We cannot exclude that the selection bias introduced by the dropout might have affected the study results. Furthermore, as the TR group underwent intervention during the COVID-19 pandemic, we cannot exclude that their physical and psychological outcomes might have been compromised by the COVID-19 restrictions and the need for social isolation (45, 46).

Additional limitations are intrinsic to the chosen study design. First, as our intervention was individually tailored, this might result in reduced generalizability and increased complexity of replication (47). Second, although our primary and most of our secondary outcomes were based on PROMs, we cannot exclude that knowing their group allocation might have affected the pwMS perception of effectiveness.

Third, due to safety concerns, we did not include people with severe motor disabilities. Although these subjects are the ideal candidates for home-based interventions, the implementation of an active exercise program (like the TR program adopted in the current study) in these cases is difficult, with the risk of injury increasing with the level of motor disability and the growing need to rely on passive exercise.

As follow-up was limited to 6 weeks, we cannot speculate about the duration of the observed improvements over time. Finally, the COVID-19 pandemic affected our enrollment and randomization processes. Despite this, the two study groups were well-balanced for baseline features, and, as an additional precaution, baseline demographic and clinical characteristics were included in the statistical analysis as covariates of no interest.

In conclusion, our tailored rehabilitation intervention effectively improved QoL, fatigue, balance, and cognition in pwMS. More importantly, similar outcomes were achieved in the TR and SR groups at both the group and individual levels, suggesting the validity of the TR approach in a population of MS patients with moderate disability.

From a clinical perspective, our findings support the application of supervised physical therapy to achieve multi-domain improvements that, although moderate in terms of effect size, appear to exert a meaningful impact at the individual level in up to 50% of the exposed population. From a research standpoint, our results would benefit from further confirmation across a wider range of disability levels and longer follow-ups to establish the persistence of the observed clinical improvements over time.

## Data availability statement

The raw data supporting the conclusions of this article will be made available by the authors, without undue reservation.

## Ethics statement

The studies involving humans were approved by Ethics Committee of Sapienza University of Rome (5416 21/11/2019). The studies were conducted in accordance with the local legislation and institutional requirements. The participants provided their written informed consent to participate in this study.

## Author contributions

MP: Conceptualization, Data curation, Formal analysis, Investigation, Methodology, Validation, Writing – original draft, Writing – review & editing. NP: Conceptualization, Data curation, Formal analysis, Funding acquisition, Investigation, Methodology, Project administration, Supervision, Writing – original draft, Writing – review & editing. GS: Conceptualization, Data curation, Investigation, Methodology, Project administration, Writing – original draft, Writing – review & editing. IR: Data curation, Investigation, Methodology, Writing – review & editing. CL: Data curation, Investigation, Methodology, Writing – review & editing. VB: Data curation, Investigation, Methodology, Writing – review & editing. FF: Data curation, Investigation, Methodology, Writing – review & editing. AI: Data curation, Investigation, Methodology, Writing – review & editing. SR: Data curation, Investigation, Methodology, Writing – review & editing. GB: Investigation, Methodology, Writing – review & editing. CP: Conceptualization, Data curation, Funding acquisition, Investigation, Methodology, Project administration, Supervision, Writing – original draft, Writing – review & editing.
